# A systematic review and meta-analysis of the effectiveness of social support on turnover intention in clinical nurses

**DOI:** 10.3389/fpubh.2024.1393024

**Published:** 2024-06-06

**Authors:** Yan Chen, Xiang Zhou, Xue Bai, Beibei Liu, Fengzhi Chen, Lixia Chang, Hongli Liu

**Affiliations:** ^1^Department of Critical Care Medicine, Shandong Provincial Hospital Affiliated to Shandong First Medical University, Jinan, China; ^2^School of Sociology and Political Science, Shanghai University, Shanghai, China

**Keywords:** nurses, social support, turnover intention (TI), meta-analysis, moderators

## Abstract

**Background:**

Nurse turnover has become a salient issue in healthcare system worldwide and seriously compromises patient outcomes. Social support is considered an effective contributor to alleviate nurse turnover intention (TI). However, the degree of correlation between social support and nurse TI remains elusive.

**Aims:**

This study aims to evaluate the strength of the effectiveness of social support on TI among nurses as well as its potential moderators.

**Design:**

This systematic review and meta-analysis followed the Preferred Reporting Items for Systematic Reviews and Meta-Analyses.

**Methods:**

To obtained qualified studies, two researchers searched Embase, PubMed, Web of science, CINAHL, CNKI, WanFang, and Chinese Medical Journal Full Text Database from inception to January 6, 2024. Meta-analysis, publication bias, and sensitivity analysis were carried out on the included studies using CMA 3.0 software, and the moderating effect was verified through meta-analysis of variance (ANOVA).

**Results:**

A total of 38 studies were obtained, involving 63,989 clinical nurses. The comprehensive effect size of the random effect model showed a significant medium negative correlation between social support and TI among nurses (*p* < 0.001). The sample size and TI measurement tools significantly moderated the correlation between social support and TI (*p* < 0.050). However, nurse department, gender, data collection time, and social support measurement tools did not moderate the correlation between the two variables.

**Conclusion:**

Social support is negatively associated with TI in nurses. Nursing administrators and the medical community should fully recognize the importance of social support for nurses and take corresponding measures to enhance it, thereby reducing TI and ensuring the stability of the nursing team.

## 1 Introduction

The demand for nurses is growing rapidly due to the extended life expectancy, aging populations, and increased need for high-quality healthcare services (1–3). By 2035, the aggregate need for nurses is likely to reach 12.9 million (4). In reality, however, there is a significant gap between the supply and demand of nursing staff. Almost all healthcare systems worldwide are facing a nursing shortage and it is expected that by 2030, there will be a shortfall of 10 million nurses (5). The shortage of nursing staff will make a series of negative impacts including but not limited to increasing the hospital infection rate (6), medical error rate (7), patient readmission rate (8), pressure ulcer incidence (7), and even mortality (9), which ultimately compromises the patient safety and quality of medical services.

Nursing shortage is an ongoing issue in health organizations and researchers (10, 11). There is a plenty of factors contributing to the shortage of nursing staff, among which the high nursing turnover rate is considered one of the major ones (12). Frequent turnover behaviors may reduce the organizational efficiency, lead to emotional instability and lax behavior among other employees in the organization, and increase hospital investment in nurse training (13). In the case of a shortage of nurses, it is imperative that nurse managers plan effective retention strategies based on the reasons for staff resignation (14). Turnover intention (TI) refers to the tendency of employees to leave their current job positions and seek other job opportunities (15), which is considered an important cognitive process before turnover behavior occurs, and hence, it is the best and reliable antecedent variable for predicting turnover behavior (16). The higher the TI, the greater the likelihood of turnover behavior occurring (17). Moreover, TI can also subside the work enthusiasm and stability of nurses, and impair the nursing service quality (15).

In recent years, scholars worldwide are dedicated to exploring the factors that affect nurse TI, and determine social support as one of the psychological and social factors beneficial for weakening nurse TI (18, 19). Social support is defined as providing assistance and protection to others, especially individuals (20), including tangible economic assistance and intangible emotional assistance (21). For nurses, social support from supervisors and colleagues is an important source of perceived social support (22, 23). Nurses often face workplace stress in clinical work, including high workloads, complex patient situations, management’s leadership styles, role conflicts, and workplace aggression (24, 25). The buffering model of social support suggests that an effective social support network can alleviate the negative psychological consequences of stress (26) and is also an important protective factor in alleviating employee turnover (23).

Numerous studies have been conducted on the correlation between social support and TI in nurses. However, there are considerable divergences in the results of the existing studies, especially the degree of correlation between the two variables. For instance, Lei et al. (27) conducted a survey on 82 female emergency department nurses using the Social Support Rating Scale (SSRS) and Turnover Intention Questionnaire (TIQ), and found that the correlation coefficient between social support and TI was −0.711. Yu and Gui (28) measured 445 nurses from emergency department (93.03% female) using the Perceived Social Support Scale (PSSS) and a single item in 2020, and found that the *r* value of the correlation between two variables was −0.478. However, Gülcan (29) evaluated 183 clinical nurses (86.3% female) using a 6-item and 3-item questionnaire, and found that the *r* value of the correlation between social support and TI was −0.154. In addition to the different degrees of correlation, the correlation between supervisor support and TI, as well as the correlation between colleague support and TI, were also reported in different directions. By using a 4-item questionnaire and a 3-item questionnaire, Adriaenssens and Van Bogaert (30) concluded *r* values of 0.313 and 0.039 for the correlation between supervisor support and colleague support with TI among nurses, respectively. Galletta et al. (31) used an adapted version of the Perceived Organizational Support Questionnaire and a two-item questionnaire to measure the correlation between supervisor support and TI, yielding a *r* value of −0.187 for the two variables. In the study of Pisarski et al. (22), a negative correlation (*r* = −0.296) was also reported between colleague support and TI. The differences in the degree and direction of correlation between social support and TI among nurses may be attributed to the differences in the study sample, study design, and measurement tools. Therefore, it is necessary to synthesize the existing research results to verify the correlation between social support and TI among nurses and to further analyze the moderating variables that affect the correlation between the two.

Regarding the study sample, existing studies involve nurses from different departments (such as emergency department, operating room, or departments that have not been clearly reported) and nurses of different genders. The specific work undertaken by nurses in different departments may affect the TI to varying degrees. For example, as the front-line staff about hospital systems, nurses from emergency department face challenging working conditions due to casualty incidents and potentially violent situations (32, 33). Moreover, the work of the emergency department is characterized by a wide range of pathologies and a broad variety of emergencies, leading to higher nursing requirements (34). In contrast, nurses from operating room are responsible for sterility, equipment, infection, complication control, and biological specimen management during surgery, while also adapting to the different personalities and surgical techniques of different surgeons (35, 36). A previous study showed that the TI of nurses from emergency department and ICU was higher than that of general ward nurses (37). Kim and Park (38) pointed out that pediatric nurses were under greater pressure and had higher TI than general ward nurses. Additionally, there are also gender differences in the TI and perceived social support of nurses. For example, Ma et al. (32) and Zhao et al. (39) reported no significant difference in TI scores of nurses of different genders. Conversely, Xu et al. (19) found that the TI score of male nurses was significantly higher than that of female nurses. Therefore, we propose the following hypothesis: the department and gender of nurses may affect the correlation between social support and TI.

In terms of study design, there are also differences in the sample size and data collection time of existing studies. Sample size is a key parameter for the calculation of comprehensive correlation coefficients (40). In a study with a small sample size, the magnitude of the correlation is quite unstable (41). Previous studies on the correlation between social support and TI showed significant differences in the sample size, ranging from 82 (27) to 16,052 (42). Furthermore, the impact of data collection time on the correlation between the two variables should not be ignored. In particular, the COVID-19 pandemic has increased the demand and workload for nurses. The uncertainty and mortality of diseases also put tremendous psychological pressure on nurses. Mirzaei et al. (43) found that the intense work pressure during the COVID-19 shaped the work attitude of nurses, resulting in stronger TI among nurses. A literature review of 43 studies also revealed a significant increase in TI among nurses following the COVID-19 pandemic (44). Thus, the hypothesis of this study is as follows: sample size and data collection time may be potential moderators of the correlation between social support and TI.

In addition to the variables mentioned above, existing studies have included different measurement tools for social support and TI, which may also moderate the correlation between these two variables. Social Support Rating Scale (SSRS) (18, 27), Perceived Social Support Scale (PSSS) (19, 28), and multiple item measurement questionnaires (43, 45) were mainly used to measure the perceived social support of clinical nurses. Similarly, there are several different measurement tools for TI, such as turnover intention questionnaire (TIQ, 1982) (18, 39), Turnover Intention Scale (TIS) (46), and TIQ (2015) (19). Different measurement tools contain different contents. Therefore, the correlation between social support and TI among nurses may be influenced by the measurement tools.

Given the importance of maintaining professional stability in nurses and the lack of systematic meta-analyses that assess the relationship of social support with nurses’ TI, this study aimed to analyze the strength of the effectiveness of social support on the nurses’ TI as well as its potential moderators. Specifically, this study (a) calculated the overall effect size of the relationship between social support and nurses’ TI and (b) examined whether the relationship is moderated by nurse’s department, sex, sample sizes, data collection time, social support measurement tools, and TI measurement tools. This study enables nursing administrators and the medical field to pay more attention to nurses’ social support and take corresponding measures to improve it, intending to reduce nurses’ TI and promote a more stable nursing team.

## 2 Methods

### 2.1 Study design

This study was designed and written following the Preferred Reporting Items for Systematic Reviews and Meta-Analyses (PRISMA) (47). This agreement has been registered in the International Prospective Register of Systematic Reviews (PROSPERO; number: CRD42023476373).

### 2.2 Search strategy

A comprehensive search was conducted on CNKI, Wanfang, Chinese Medical Journal Full Text Database, PubMed, Web of Science, Embase, and CINAHL databases to obtain relevant literature on clinical nurses’ social support and TI from the inception of the database to January 6, 2024. The search terms included “nurses,” “social support,” and “turnover intention.” The search terms for nurses included “nurse” and “nursers.” The search term for social support included “social support.” The search terms for TI included “turnover intention,” “turnover to quit,” “turnover to leave,” and “resignation intention.” To further expand the search scope, we checked the list of references included in the literature. The detailed search formula can be found in Supplementary material 1.

### 2.3 Inclusion criteria

The retrieved literature was screened by two researchers based on the following inclusion and exclusion criteria. Inclusion criteria: (1) considering the availability of data on correlations between the two variables, study types were limited to cross-sectional studies and longitudinal studies reporting multiple cross-sections; (2) published in English or Chinese; (3) participants are clinical nurses, regardless of department; (4) reporting on social support and TI or calculating the Pearson’s correlation coefficient based on existing data; (5) applied clear tools for measuring social support and TI, including, but not limited to, PSSS (1988) and SSRS (1986) for social support and TIQ (1982) and TIS (1991) for TI; (6) the type of literature is limited to journal article. Exclusion criteria: (1) studies with the same data and repeated publications; (2) studies with data errors, such as those where the sample size or the correlation coefficient reported different data.

### 2.4 Data extraction

After literature screening, the two researchers read the entire text to extract data. The extracted information was as follows: author, year, country, publication type, sample size, sample gender, department, sample collection time, social support measurement tool, TI measurement tool, as well as Pearson correlation coefficient between social support and TI. If several different samples were investigated in the same study, they were extracted separately. Any disputes during the data extraction process were discussed and decided by two researchers.

### 2.5 Quality assessment tool

The “Joanna Briggs Report Epidemic Data Research Institute Key Assessment Checklist” (48) was used for quality evaluation. This checklist consists of 9 items, each of which includes four answers (“yes,” “no,” “unclear,” and “not applicable”). If the answer is “yes,” 1 point will be given; If the answer is “no,” “unclear,” or “not applicable,” zero point will be scored. The total score of the scale is 9, and the higher the score, the better the quality of the study.

### 2.6 Statistical analysis

Pearson correlation coefficient was used to calculate the magnitude of correlation between social support and TI. Firstly, we used the formula Fisher’s *Z* = 0.5 ln [(1 + *r*)/(1-*r*)] to convert the value of *r* to Fisher’s *Z*. Then, based on the sample size, the obtained values were weighted using the formula SEz = $\sqrt{1/\left(n-3\right)}$ and the reciprocal of the variance of the correlation coefficient. Finally, the formula Summary *r* = ($e^{2z}-1$)/($e^{2z}+1$) was used to convert all values into *r* to evaluate the correlation between social support and TI. According to the study of Gignac and Szodorai (49), *r* = 0.10, *r* = 0.20, and *r* = 0.30 are indicative of relatively small, medium, and relatively large correlations, respectively. Heterogeneity was determined using Cochran’s *Q*-test and *I^2^* statistics (50). The random effect model did not assume a common potential effect size for all included studies (51), making the random effect model more suitable for current analysis than the fixed effect model. In addition, a meta-analysis of variance (ANOVA) was used to test the possible moderating variable between social support and TI. Inter- and intra-group comparisons were performed using *Q*-test. Funnel plots, Begg test (52), and Egger test (53) were applied to evaluate the publication bias. When the funnel plot is symmetrical at both ends and the *p*-values >0.05 for both Begg test and Egger test, it is considered that there is no publication bias. Simultaneously, sensitivity analysis was conducted to test the robustness of the results. All statistical analyses of this study were conducted using the software Comprehensive Meta-Analysis (CMA), version 3.0.

## 3 Results

### 3.1 Study characteristics and quality assessment

**Figure 1:**
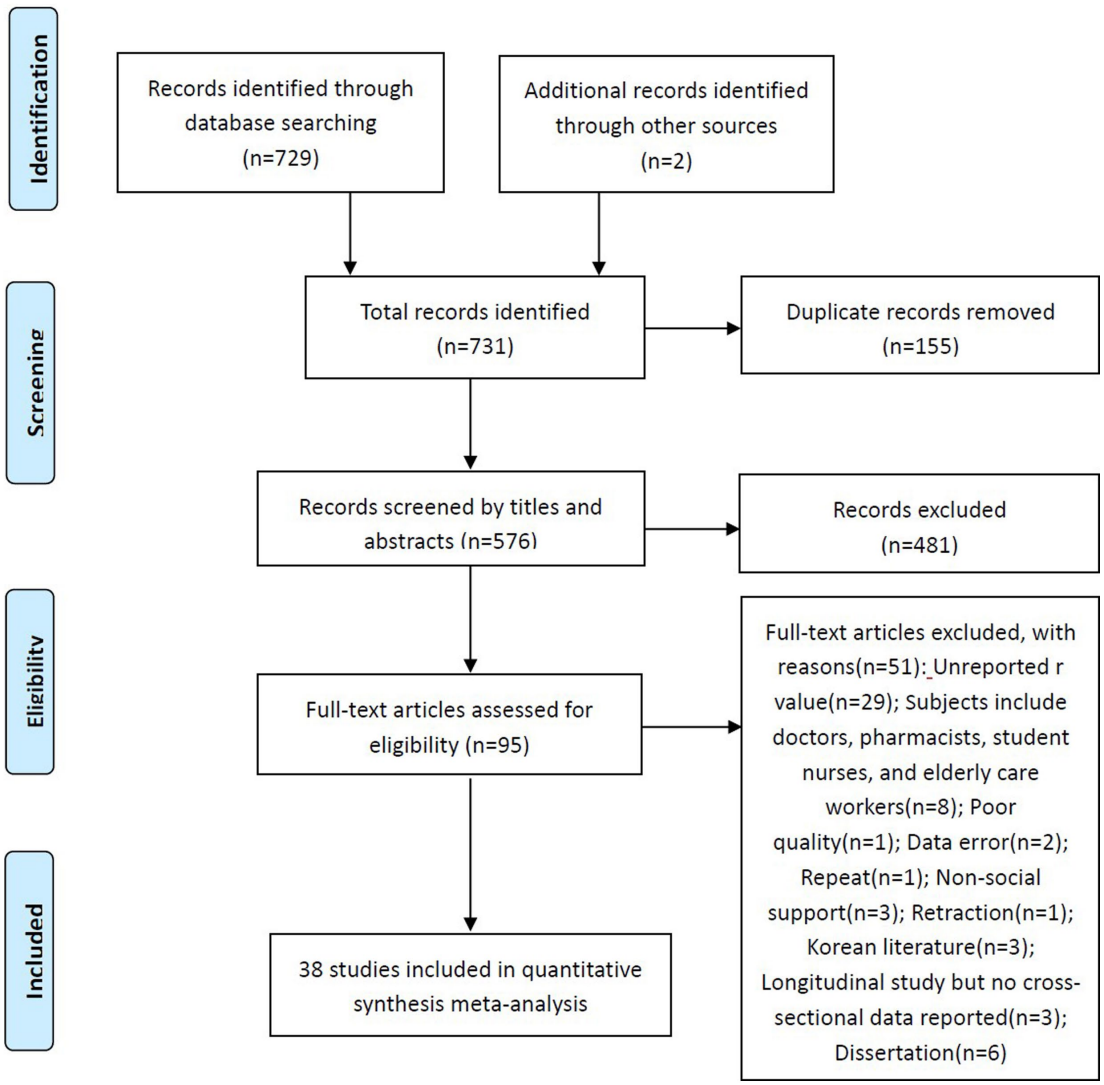
The process of literature screening.

A total of 731 records (Embase 62, PubMed 34, Web of Science 426, CINAHL 59, CNKI 94, WanFang 51, Chinese Medical Journal Full Text Database 3, and other sources 2) were preliminarily searched in this study (Figure 1). After duplicate removal, 576 studies were obtained. Thereafter, we read the titles and abstracts and reviewed the full text of 95 studies, obtaining 39 eligible studies. Finally, after discussion between two researchers, studies with poor quality will be excluded. A total of 38 studies were obtained (Table 1), with a total sample size of 63,989. Zhou and Wang (76) reported the correlation between social support and TI among nurses in secondary and tertiary hospitals. van der Heijden et al. (23) and Gabel Shemueli et al. (46) both reported the correlation between two variables in nurses from different countries. In the study of Tei-Tominaga et al. (45), the correlation between social support and TI among nurses at different birth stages was reported. For the quality assessment of the included studies, 10 studies scored 6 points, 14 studies scored 7 points, 13 studies scored 8 points, and only 1 study scored 9 points. The detailed quality assessment of included studies can be found in Supplementary material 2.

**Table 1:** Characteristics of 38 included studies.

Study	Sample size (women/men)	Age (mean ± sd/range)	Country	Department	Study type	Social support measurement tool	Turnover intention measurement tool
Schmieder and Smith (54)	191 (183/8)	35	USA	No report	Cross-sectional	13-item scale developed by House and Wells (55)	Three items from the Michigan Organizational Assessment Questionnaire [Seashore et al. (56)]
Baba et al. (57)	119 (108/11)	37.29 ± 8.87	Caribbean	No report	Cross-sectional	10 items taken from House and Wells (55)	Three items questionnaire adopted from Mobley (58)
Pisarski et al. (22)	1,257 (1,113/144)	Public hospitals: 36 Private hospitals: 39	Australia	No report	Cross-sectional	A scale adapted from Caplan et al. (59)	The scales developed by Caplan et al. (59)
Widerszal-Bazyl et al. (42)	16,052 (16,052/0)	39.4 ± 8.8	Europe	No report	Cross-sectional	Developed by Van der Heijden (60)	Three items questionnaire adopted from Mobley (58)
van der Heijden et al. (23)	Belgium: 1,686 (1,686/0) Germany: 2,048 (2,048/0) Finland: 1,724 (1,724/0) France: 2,182 (2,182/0) Italy: 3,308 (3,308/0) Netherlands: 2,127 (2,127/0) Poland: 3,089 (3,089/0) Slovakia: 1,360 (1,360/0)	Belgium: 37.7 ± 8.96 Germany: 38.01 ± 9.2 Finland: 42.14 ± 10.04 France: 38.65 ± 9.31 Italy: 38.23 ± 7.56 Netherlands: 37.93 ± 9.45 Poland: 38.71 ± 7.21 Slovakia: 40.37 ± 8.26	Belgium Germany Finland France Italy Netherlands Poland Slovakia	No report	Cross-sectional	Four items [Van der Heijden (61)]	One item
Adriaenssens et al. (62)	254 (140/114)	37.61 ± 8.82	Belgium	Emergency	Cross-sectional	4 items (Leiden Quality of Work Questionnaire for Nurses)	3 items (Leiden Quality of Work Questionnaire for Nurses)
Galletta et al. (31)	1,240 (1,010/230)	Women: 36.95 ± 7.91 Men: 37.31 ± 8.19	Italy	No report	Cross-sectional	The adapted of the survey of perceived organizational support	Two items adapted from Hom et al. (63)
He and Sun (64)	210 (No report)	28.27 ± 4.27	China	Emergency	Cross-sectional	SSRS [Xiao (65)]	Turnover Intention Questionnaire [Brough and Frame (66)]
Fang et al. (67)	194 (No report)	35.82 ± 9.5	China	No report	Cross-sectional	PSSS [Zimet et al. (68)]	TIQ [Michaels and Spector (69)]
Lei et al. (27)	82 (82/0)	No report	China	Emergency	Cross-sectional	SSRS [Xiao (65)]	TIQ [Michaels and Spector (69)]
van Dam et al. (70)	461 (No report)	41.9 ± 9.43	Netherlands	Intensive care	Cross-sectional	van Veldhoven et al. (71)	Turnover intention scale [van Dam (72)]
Cai et al. (73)	133 (128/5)	21–25	China	No report	Cross-sectional	PSSS [Zimet et al. (68)]	TIQ [Michaels and Spector (69)]
Wu et al. (74)	632 (617/15)	27.2 ± 4.34	China	Multiple departments	Cross-sectional	SSRS [Xiao (65)]	TIQ [Michaels and Spector (69)]
Zheng et al. (75)	858 (850/8)	34.45 ± 6.5	China	Multiple departments	Cross-sectional	PSSS [Zimet et al. (68)]	TIQ [Michaels and Spector (69)]
Zhou and Wang (76)	Secondary Hospital: 353 (353/0) Tertiary Hospital: 438 (438/0)	No report	China	Emergency	Cross-sectional	SSRS [Xiao et al. (65)]	TIQ [Michaels and Spector (69)]
Gabel Shemueli et al. (46)	Uruguay: 316 (299/17) Spain: 502 (458/44)	Uruguay: 40.3 ± 9.78 Spain: 44 ± 10.8	Uruguay Spain	No report	Cross-sectional	Dolan et al. (77)	TIS [Arsenault et al. (78)]
Adriaenssens and Van Bogaert (30)	318 (188/130)	45.7	Belgium	Multiple departments	Cross-sectional	4 items (Leiden Quality of Work Questionnaire for Nurses)	3 items (Leiden Quality of Work Questionnaire for Nurses)
Chen et al. (79)	1,305 (1,247/58)	35.89 ± 6.36	China	Operating theatre	Cross-sectional	SSRS [Xiao (65)]	TIQ [Michaels and Spector (69)]
Tei-Tominaga et al. (45)	Born in 1950–1964: 673 (673/0) Born in 1965–1979: 1,912 (1,912/0) Born during the 1980s: 1,786 (1,786/0) Boen after 1990: 693 (693/0)	Born in 1950–1964: 54.23 ± 3.13 Born in 1965–1979: 41.38 ± 4.24 Born during the 1980s: 29.91 ± 7.1 Boen after 1990: 22.99 ± 0.86	Japan	No report	Cross-sectional	Three-item original scale, which was developed by the researchers after referring to previous studies	Six-item scale
Xie et al. (80)	175 (174/1)	No report	China	Pediatric	Cross-sectional	SSRS [Xiao (65)]	TIQ [Michaels and Spector (69)]
Zhu and Qin (81)	282 (259/23)	28.01 ± 4.04	China	No report	Cross-sectional	SSRS [Xiao (65)]	Turnover intention scale [Zhang (82)]
Huang et al. (83)	370 (348/22)	No report	China	No report	Cross-sectional	The Social Network Model Scale	Departure Disposition Scale [Richard and Johnson (84)]
Wang et al. (85)	2,345 (2,280/65)	29.74 ± 7.41	China	Multiple departments	Cross-sectional	SSRS [Xiao (65)]	TIQ [Michaels and Spector (69)]
Yeh et al. (86)	198 (188/10)	No report	China	No report	Cross-sectional	The Chinese version of the Job Content Questionnaire (C–JCQ) [Cheng et al. (87)]	Four questions [Mobley (58)]
Cao et al. (88)	361 (298/63)	22.38 ± 1.23	China	Multiple departments	Cross-sectional	PSSS [Zimet et al. (68)]	Turnover intention scale [Lee and Lee (89)]
Cole et al. (90)	111 (No report)	No report	USA	No report	Cross-sectional	Four items	Two items
Hognestad Haaland et al. (91)	2,946 (2,661/285)	No report	Norway	Multiple departments	Cross-sectional	A three-item scale developed by van der Heijden (60)	Three items
Meng et al. (92)	177 (0/177)	20–45	China	Multiple departments	Cross-sectional	PIS [Liu et al. (93)]	TIQ [Michaels and Spector (69)]
Mirzaei et al. (43)	479 (295/184)	33.48 ± 6.77	Iran	Multiple departments	Cross-sectional	Social support scale (8 items)	Turnover Intention Questionnaire [Kim and Leung (94)]
Modaresnezhad et al. (95)	1,080 (No report)	No report	USA	Multiple departments	Cross-sectional	Finley et al. (96)	Turnover Intention [Price (97)]
Zhao et al. (39)	296 (272/24)	No report	China	Multiple departments	Cross-sectional	SSRS [Xiao (65)]	TIQ [Michaels and Spector (69)]
Zhang et al. (98)	594 (587/7)	30 ± 7.6	China	No report	Cross-sectional	SSRS [Xiao (65)]	TIQ [Michaels and Spector (69)]
Wu et al. (99)	118 (105/13)	No report	China	Operating theatre	Cross-sectional	Social Support Scale [Liu et al. (100)]	Cole and Bruch (101)
Xiao et al. (18)	4,865 (4,738/127)	No report	China	Multiple departments	Cross-sectional	SSRS [Xiao (65)]	TIQ [Michaels and Spector (69)]
Yu and Gui (28)	445 (414/31)	30.74 ± 6.81	China	Emergency	Cross-sectional	PSSS [Zimet et al. (68)]	One item
Zhang et al. (40)	488 (486/2)	≥18	China	Multiple departments	Cross-sectional	Occupational Stress Inventory (1998)	TIQ [Michaels and Spector (69)]
Li (102)	96 (87/9)	31.12 ± 3.59	China	Emergency	Cross-sectional	SSRS [Xiao (65)]	TIQ [Michaels and Spector (69)]
Xu et al. (19)	1,060 (985/75)	32.94 ± 7.876	China	Operating theatre	Cross-sectional	PSSS [Zimet et al. (68)]	The turnover intention questionnaire [Lee et al. (103)]

SSRS, Social Support Rating Scale; PSSS, Perceived Social Support Scale; PIS, Professional Identity Scale; TIQ, Turnover Intention Questionnaire; TIS, The Turnover Intention Scale.

### 3.2 Effect size and heterogeneity

#### 3.2.1 The summary correlation between social support and TI

**Figure 2:**
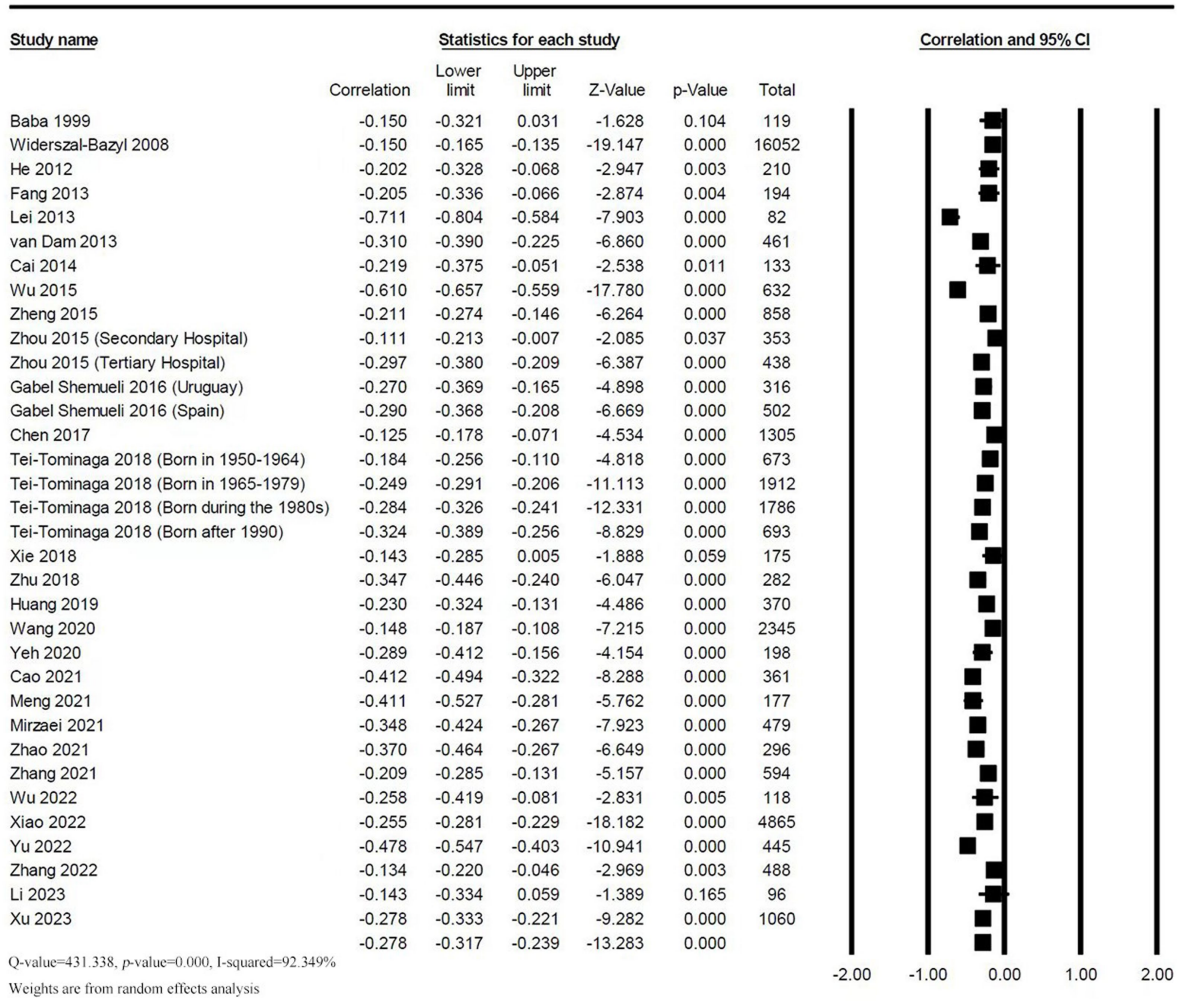
Forest plot of the correlation between social support and TI.

Data on a total of 39,068 clinical nurses was included in 34 effect sizes from 29 studies. Heterogeneity test results showed a high heterogeneity among included studies (*Q* = 431.338, *p* < 0.001, *I^2^* = 92.349%). As shown in Figure 2, the random effect model indicated a significant negative correlation between social support and TI (*r* = −0.278, 95% *CI*: −0.317, −0.239, *p* < 0.001).

#### 3.2.2 The summary correlation between supervisor support and TI

**Figure 3:**
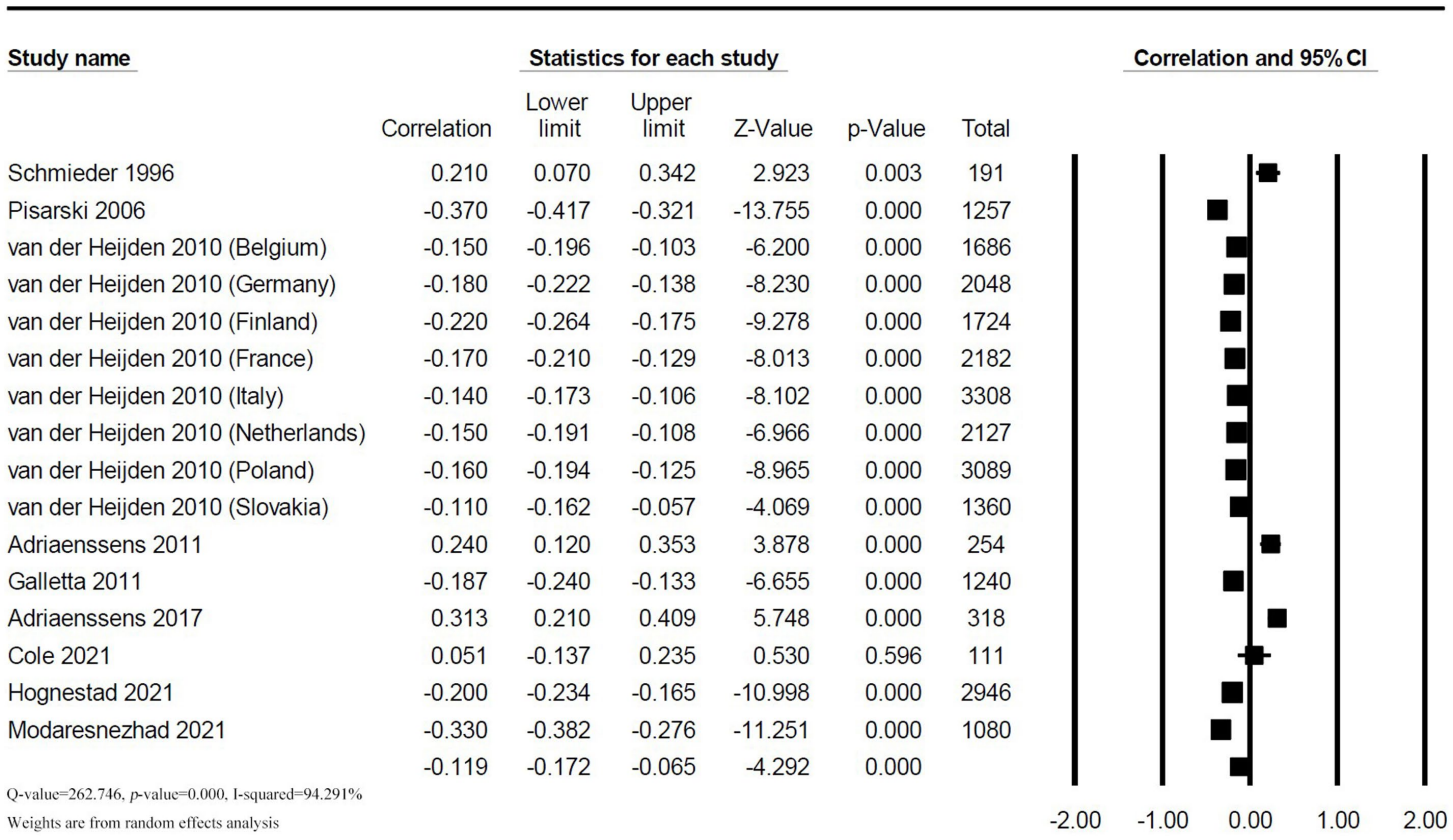
Forest plot of the correlation between supervisor support and TI.

The correlation between supervisor support and TI was reported in 9 studies with 16 effect sizes. Heterogeneity test found a high heterogeneity (*Q* = 262.746; *p* < 0.001; *I^2^* = 94.291%). The random effect model showed a low negative correlation between supervisor support and TI among nurses (*r* = −0.119, 95% *CI*: −0.172, −0.065, *p* < 0.001), as shown in Figure 3.

#### 3.2.3 The summary correlation between colleague support and TI

**Figure 4:**
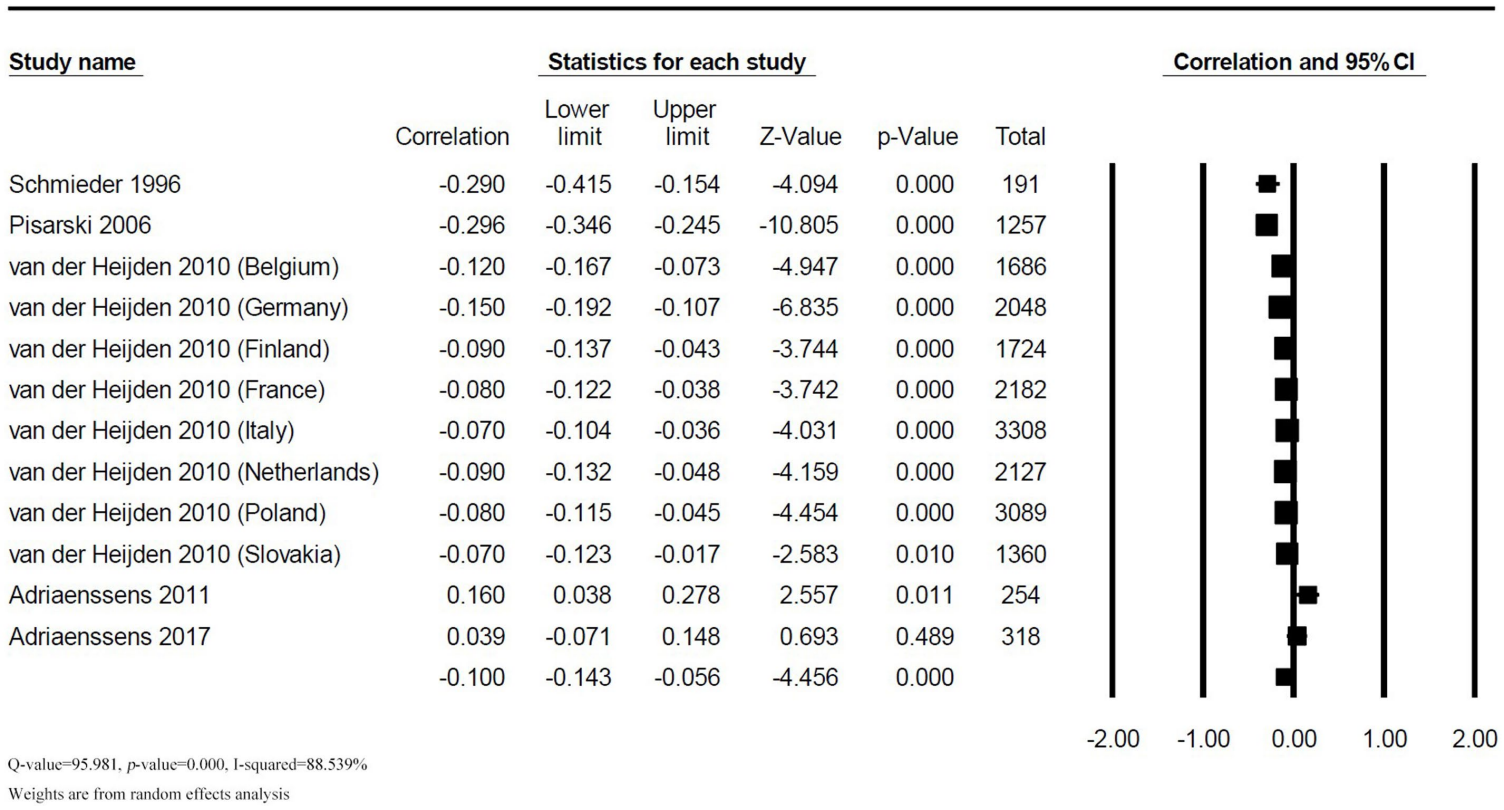
Forest plot of the correlation between colleague support and TI.

The correlation between colleague support and TI was reported in 5 studies with 12 effect sizes. There was a high heterogeneity among the combined results (*Q* = 95.981, *p* < 0.001, *I^2^* = 88.539%). The random effect model results revealed a significant negative correlation between colleague support and TI among nurses (*r* = −0.100, 95% *CI*: −0.143, −0.056, *p* < 0.001), as shown in Figure 4.

#### 3.2.4 Publication bias and sensitivity analysis

**Figure 5:**
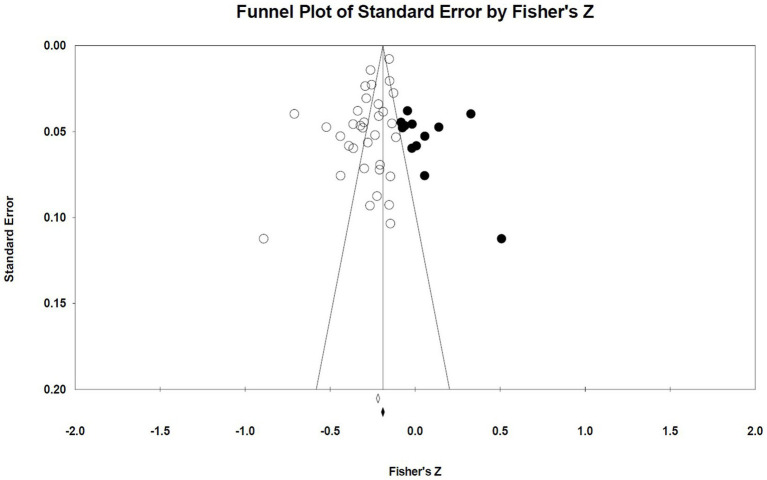
Funnel plot of the correlation between social support and TI.

**Figure 6:**
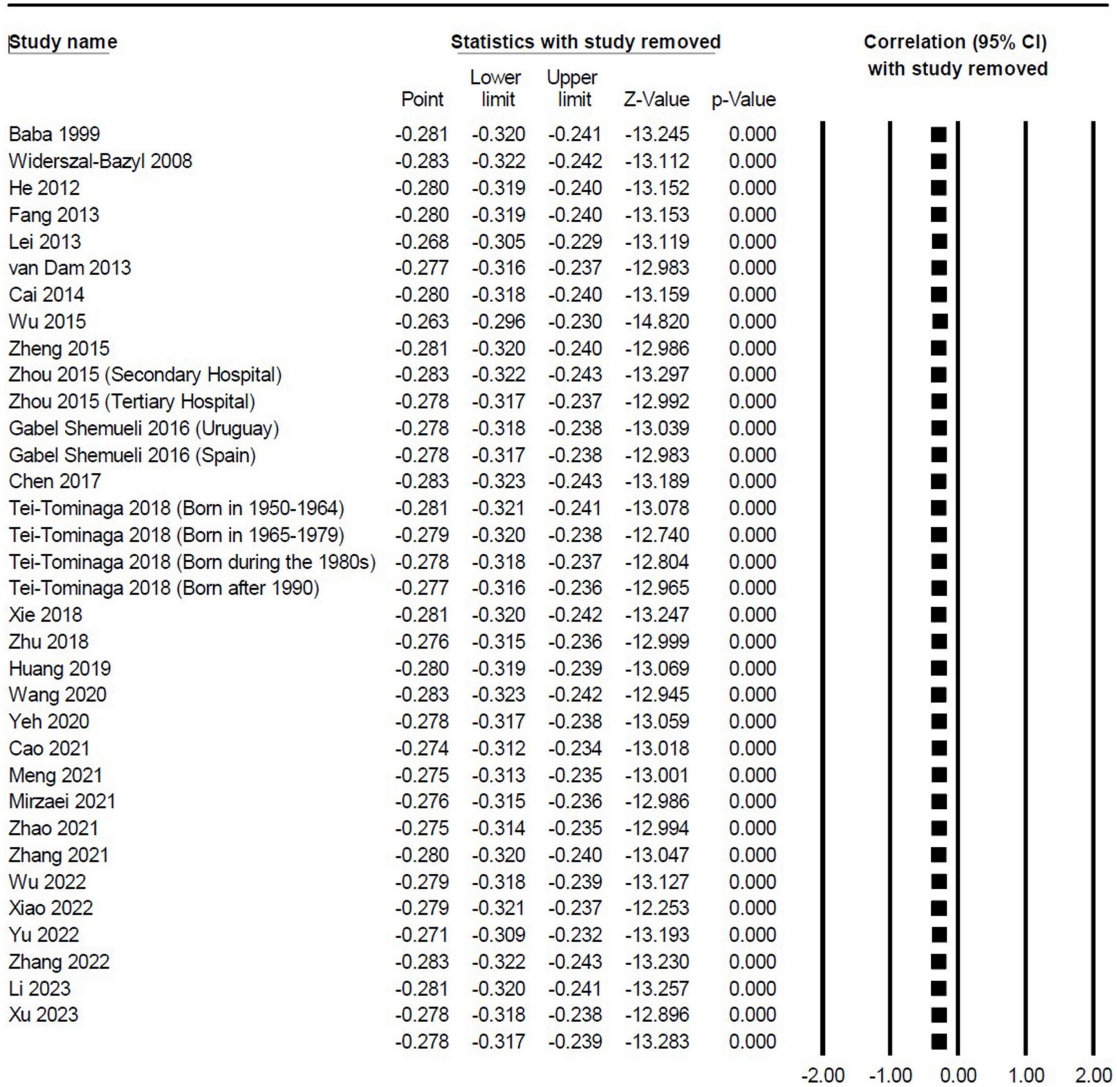
Sensitivity analysis of the correlation between social support and TI.

This meta-analysis used funnel plot, Begg’s test, and Egger liner regression to evaluated the publication bias. The effect sizes of studies included in the meta-analysis were mostly distributed on the left side of the funnel plot, suggesting a high possibility of publication bias. The results of Begg test did not show publication bias (*p* = 0.988). However, the Egger liner regression results showed significant publication bias (*t* = 3.312, *p* = 0.002). When quantifying the potential effect of small study bias on overall effect size using the trim-and-fill method, 12 studies with missing hypotheses were added, with an estimated effect size of −0.195 (95% *CI*: −0.238, −0.152), indicating a significant negative correlation between social support and TI (Figure 5). Moreover, sensitivity analysis of one-by-one elimination showed a stable effect size between social support and TI. Therefore, it was suggested that the results drawn from the meta-analysis were reliable (Figure 6).

Meanwhile, the funnel plot of the correlation between supervisor support and TI, as well as the funnel plot of the correlation between colleague support and TI, showed asymmetry, indicating potential publication bias. After a correction for the potential effect of small study bias on overall effect size using the trim-and-fill method, 5 studies with missing hypotheses were added in the correlation between supervisor support and TI, with an estimated effect size of −0.199 (95% *CI*: −0.255, −0.141); 2 studies with missing hypotheses were added in the correlation between colleague support and TI, with an estimated effect size of −0.123 (95% *CI*: −0.167, −0.078). The adjusted funnel plots can be found in the Supplementary material 3.

### 3.3 Moderator analyses

This study used a meta-analysis of variance to test the moderating effects of six variables including nurse department, gender, sample size, data collection time, social support measurement tool, and TI measurement tool. The results showed that the sample size and TI measurement tool moderated the correlation between social support and TI (sample size: *W_QBET_* = 5.044, *p* = 0.025, TI measurement tool: *W_QBET_* = 70.714, *p* < 0.001). Specifically, compared to studies with a sample size >1,000, studies with a sample size ≤1,000 reported a stronger correlation between social support and TI (studies with a sample size >1,000: *r* = −0.213, 95% *CI*: −0.262, −0.162, *p* < 0.001; studies with a sample size ≤1,000: *r* = −0.299, 95% *CI*: −0.353, −0.242, *p* < 0.001). Regarding the TI measurement tool, studies used other measurement tools had the largest effect on social support and TI (*r* = −0.329, 95% *CI*: −0.380, −0.277, *p* < 0.001), while studies used the three-item questionnaire (1977) had the smallest effect on social support and TI (*r* = −0.150, 95% *CI*: −0.165, −0.135, *p* < 0.001). However, the correlation between social support and TI was not significantly moderated by nurse department, gender, data collection time, and social support measurement tool (*p* > 0.050) (Table 2).

**Table 2:** Social support and TI: univariate analysis of variance for moderator variables.

Moderators	*Q_BET_*	*k*	*N*	*r*	95% CI		*Q_W_*	*I^2^*
Nurse’s department								
Pediatric	5.497	1	175	−0.143	−0.285	0.005	0	0%
Emergency nurse		6	1,624	−0.340	−0.491	−0.169	64.147	92.205%
Operating theatre		3	2,483	−0.215	−0.333	−0.091	15.395	87.009%
Intensive care		1	461	−0.310	−0.390	−0.225	0	0%
Unreported or multiple departments		23	34,325	−0.278	−0.322	−0.232	324.797	93.227%
Sex								
Female	6.140	11	23,510	−0.242	−0.299	−0.184	113.399	91.182%
Males		1	177	−0.411	−0.527	−0.281	0	0%
Mixed		19	14,516	−0.295	−0.354	−0.234	238.060	92.439%
No report		3	865	−0.254	−0.329	−0.177	2.779	28.044%
Sample sizes								
≤1,000	5.044^*^	27	9,743	−0.299	−0.353	−0.242	230.025	88.697%
>1,000		7	29,325	−0.213	−0.262	−0.162	93.893	93.610%
Data collection time								
During the COVID-19 pandemic	1.725	7	7,424	−0.325	−0.394	−0.252	40.001	85.000%
During the non-COVID-19 pandemic		25	30,979	−0.267	−0.313	−0.220	327.834	92.679%
Unreported data collection time		2	665	−0.273	−0.519	0.016	11.680	91.438%
Social support measurement tools								
PSSS 1988	1.138	6	3,051	−0.309	−0.400	−0.212	36.891	86.447%
SSRS 1986		13	11,673	−0.290	−0.372	−0.205	230.716	94.799%
Three-item		4	5,064	−0.262	−0.308	−0.214	9.062	66.896%
Others		11	19,280	−0.257	−0.318	−0.194	64.305	84.449%
TI measurement tools								
Three items questionnaire 1977	70.714^***^	2	16,171	−0.150	−0.165	−0.135	0	0%
TIS 1991		2	818	−0.282	−0.344	−0.218	0.091	0%
TIQ 1982		15	12,854	−0.266	−0.338	−0.190	233.284	93.999%
Six-item		4	5,064	−0.262	−0.308	−0.214	9.062	66.896%
Others		11	4,161	−0.329	−0.380	−0.277	32.064	68.812%

k, number of effect sizes; N, number of samples; *Q*_BET_, between groups; Q_W_, within groups; ^*^p < 0.05, ^***^p < 0.001.

## 4 Discussion

To our knowledge, the current study is the first meta-analysis to quantitatively examine the correlation between social support and TI among nurses using correlation coefficients. The findings indicated that nurses’ perceived social support was moderately negatively correlated with TI, indicating that nurses with high perceived social support had low TI. This finding is consistent with the buffering hypothesis of social support, suggesting that social support as an effective resource can help nurses cope with work pressure, alleviate negative emotions, and reduce TI (18, 104). Given that the shortage of nursing staff is a prominent problem that is being experienced worldwide. Efforts should be made by nursing managers and researchers to understand and ameliorate the factors that lead to nurse turnover, thereby promoting nurse retention. The promotion of social support for nurses may be a measure worthy of attention.

### 4.1 The relationship between supervisor support, colleague support and TI

The current study also found that supervisor support and colleague support were negatively correlated with nurse TI. van der Heijden et al. (23) pointed out that working environment factors including social support from supervisors and colleagues could positively preventing nurses from leaving the nursing profession prematurely. Social support from supervisors could enhance the confidence of subordinates in career development (105) and contribute to building an intimate relationship between superiors and subordinates (90). Lack of job satisfaction is an important risk factor for nurse turnover, and a close superior-subordinate relationship is beneficial for organizational outcomes such as job satisfaction and happiness (106). According to the Conservation of Resources (COR) theory, social support from supervisors provides a variety of tangible and intangible resources to alleviate the turnover tendency caused by the job itself (95). Similarly, social support from colleagues was negatively correlated with nurse TI. Since nursing work requires high-quality teamwork and close colleague support (107), lack of support from colleagues can cause low-quality interpersonal relationships and eventually induce turnover behaviors (108). Moreover, compared with nurses who perceived low level of support from colleagues, nurses who perceived high level of support from colleagues were more likely to positively evaluate their team atmosphere and had a stronger sense of work identity (22). The support provided by close colleagues, including clinicians, is an important source of nurses’ perceived support. Therefore, it is crucial to facilitate effective communication and exchange between clinicians and nurses; establish a close team relationship; and ensure the work, information, and emotional support of clinicians for nurses can promote the retention of nurses.

### 4.2 Discussion of moderation effects

According to the results of the moderating effect analysis, the nursing department had no significant effect on the correlation between social support and TI, which might be related to the department distribution of the participants. In the current analysis, the vast majority of participants did not explicitly report their departments or they were involved in multiple departments, and merely a small number of studies focused on nurses in a single department (70). The number of participants may have affected the results of the analysis. Further research is necessary to effectively assess the impact of the nursing department on the correlation between social support and TI. Similarly, the moderating hypothesis about the gender of the sample was not supported. Previous studies reported that nurses of different genders did not show significant differences in perceived social support (109) and TI (24, 110). Additionally, only one study specifically focused on male nurses in the current included studies, and only four studies had more than 10% male participants. Therefore, the current study results still require further verification due to the insufficient male participants.

In this study, the moderating effect analysis of the study design found that sample size significantly affected the correlation between social support and nurse TI. Specifically, the correlation reported in studies with a sample size ≤1,000 was higher than that reported in studies with a sample size >1,000, which is consistent with the results of a previous meta-analysis (111). Existing evidence shows that there is a considerable correlation between the effect size and the sample size, that is, studies with a small sample size usually produce a larger effect size than those with a large sample size (112). The correlations between sample size and effect size can be interpreted as evidence for publication bias (112). The moderating effect test of data collection time unveiled that the data collection time had no moderating effect on the correlation between social support and TI, indicating that the correlation between social support and TI was not affected by the data collection time. Although the COVID-19 pandemic has increased the challenges and fears faced by nurses, the formation of TI is a complex, multi-stage process that starts with negative psychological responses to the current job (113, 114). As the pandemic progresses and more becomes known about the disease, nurses’ negative responses may also change. Additionally, ideological contracts may reduce the influence of fear on TI (115). Even in crisis situations, the protective effect of ideological motives remains (116).

The moderating effect analysis of the measurement tools showed that the social support measurement tools did not significantly moderate the correlation between social support and TI among nurses. The existing studies mainly rely on SSRS (1986) and PSSS (1988) to measure the social support. Although the two tools have different dimensions, their assessment contents are similar to a certain extent, such as support from family, friends, and colleagues. Given the diversity of social support measurement tools, the categorization of included studies may not fully reflect the impact of social support measurement tools on the correlation between social support and TI. In contrast, the current study found that TI measurement tools could moderate the correlation between social support and TI. Different measurement tools produce different correlation coefficients. In particular, the highest correlation coefficient was reported in other measurement tools, and the results of with TIS (1991) and TIQ (1982) were relatively close, while the lowest correlation coefficients were reported in three-item measurement questionnaires. There are significant differences in the content of the tools used to measure TI. For example, TIS (1991) (78), as a three-item scale, was more concise in content and may have stronger operability. As one of the most widely used tools for measuring TI, TIQ (1982) (69) assesses the likelihood of an individual quitting his current job, the motivation to seek other jobs, and the likelihood of obtaining other jobs, with a certain degree of stability. By comparison, the evaluation content of other one-dimensional measurement tools is limited. Therefore, among existing measurement tools, TIS (1991) may better reflect the association between social support and nurses’ TI. However, given the limited number of studies involving TIS (1991), more research is still needed to validate the current result.

### 4.3 Limitations and future research

Unlike previous studies that explore the correlation between social support and TI among nurses, the present study conducted a meta-analysis to investigate the correlation between overall social support, supervisor social support, colleague social support, and nurse TI, and further clarify the degree of correlation between variables. By synthesizing existing studies, this meta-analysis can provide more sufficient and stable evidence for implementing corresponding interventions to reduce the TI of nurses. Nevertheless, this study also has some limitations. Firstly, a few studies were published earlier in the included literature, which may not reflect the latest data on the association between social support and TI. The number of studies in some subgroups is relatively small. For instance, only one study targets ICU or pediatric nurses and the included studies mainly involve female participants, resulting in a lack of representativeness and typicality in some of our analysis results. Secondly, in terms of research types, as all included studies were cross-sectional studies, our meta-analysis only reveals the correlation between social support and TI, but fails to explain the causal relationship between the two variables. Future longitudinal study designs are warranted to elucidate the causal relationship between social support and TI among nurses. Finally, this meta-analysis only focuses on the impact of some moderators on the correlation between social support and TI among nurses. Further analysis is needed on other potential moderating variables, such as cultural background.

## 5 Conclusion

This meta-analysis indicates that social support plays a crucial role in predicting turnover intention among nurses. The institutional measures and working environment aimed at improving nurses’ perceived social support are conducive to reducing nurses’ turnover rates and ensuring the stability of the nursing team. This study’s results will help nursing administrators, hospitals, and policymakers develop corresponding strategies to maximize the perceived social support of nurses and reduce their turnover intention. Furthermore, colleague support, including mutual assistance and cooperation, is also crucial for nurse retention. Therefore, nursing managers should promote an organizational culture characterized by teamwork and integration.

## Author contributions

YC: Conceptualization, Writing – original draft, Writing – review & editing. XZ: Software, Writing – review & editing. XB: Supervision, Writing – review & editing. BL: Methodology, Writing – original draft. FC: Data curation, Writing – original draft. LC: Validation, Writing – review & editing. HL: Conceptualization, Writing – original draft.
