# Supplementary material for: A systematic review and meta-analysis of the effectiveness of social support on turnover intention in clinical nurses
Source: Front Public Health. 2024 Jun 6;12:1393024. doi: 10.3389/fpubh.2024.1393024 (PMC11187297; doi:10.3389/fpubh.2024.1393024)

Details of search strategy and search results in PubMed

| Search number | Query | Results | Time |
| --- | --- | --- | --- |
| 8 | ((("Social Support"[Mesh]) OR ((((((((((((((Support, Social[Title/Abstract]) OR (Social Care[Title/Abstract])) OR (Care, Social[Title/Abstract])) OR (Online Social Support[Title/Abstract])) OR (Online Social Supports[Title/Abstract])) OR (Social Support, Online[Title/Abstract])) OR (Social Supports, Online[Title/Abstract])) OR (Support, Online Social[Title/Abstract])) OR (Perceived Social Support[Title/Abstract])) OR (Perceived Social Supports[Title/Abstract])) OR (Social Support, Perceived[Title/Abstract])) OR (Social Supports, Perceived[Title/Abstract])) OR (Support, Perceived Social[Title/Abstract])) OR (Supports, Perceived Social[Title/Abstract]))) AND ((((((((((turnover intention[Title/Abstract]) OR (turnover to quit[Title/Abstract])) OR (turnover to leave[Title/Abstract])) OR (resignation intention[Title/Abstract])) OR (turnover intent[Title/Abstract])) OR (intention to leave[Title/Abstract])) OR (intent to leave[Title/Abstract])) OR (intention to quit[Title/Abstract])) OR (intent to quit[Title/Abstract])) OR (quit intention[Title/Abstract]))) AND (("Nurses"[Mesh]) OR (((((((Nurse[Title/Abstract]) OR (Personnel, Nursing[Title/Abstract])) OR (Nursing Personnel[Title/Abstract])) OR (Registered Nurses[Title/Abstract])) OR (Nurse, Registered[Title/Abstract])) OR (Nurses, Registered[Title/Abstract])) OR (Registered Nurse[Title/Abstract]))) | 34 | 2:23:18 |
| 7 | ("Nurses"[Mesh]) OR (((((((Nurse[Title/Abstract]) OR (Personnel, Nursing[Title/Abstract])) OR (Nursing Personnel[Title/Abstract])) OR (Registered Nurses[Title/Abstract])) OR (Nurse, Registered[Title/Abstract])) OR (Nurses, Registered[Title/Abstract])) OR (Registered Nurse[Title/Abstract])) | 219,546 | 2:22:32 |
| 6 | ((((((Nurse[Title/Abstract]) OR (Personnel, Nursing[Title/Abstract])) OR (Nursing Personnel[Title/Abstract])) OR (Registered Nurses[Title/Abstract])) OR (Nurse, Registered[Title/Abstract])) OR (Nurses, Registered[Title/Abstract])) OR (Registered Nurse[Title/Abstract]) | 155,338 | 2:17:59 |
| 5 | "Nurses"[Mesh] | 99,701 | 2:16:12 |
| 4 | (((((((((turnover intention[Title/Abstract]) OR (turnover to quit[Title/Abstract])) OR (turnover to leave[Title/Abstract])) OR (resignation intention[Title/Abstract])) OR (turnover intent[Title/Abstract])) OR (intention to leave[Title/Abstract])) OR (intent to leave[Title/Abstract])) OR (intention to quit[Title/Abstract])) OR (intent to quit[Title/Abstract])) OR (quit intention[Title/Abstract]) | 3,146 | 2:15:40 |
| 3 | ("Social Support"[Mesh]) OR ((((((((((((((Support, Social[Title/Abstract]) OR (Social Care[Title/Abstract])) OR (Care, Social[Title/Abstract])) OR (Online Social Support[Title/Abstract])) OR (Online Social Supports[Title/Abstract])) OR (Social Support, Online[Title/Abstract])) OR (Social Supports, Online[Title/Abstract])) OR (Support, Online Social[Title/Abstract])) OR (Perceived Social Support[Title/Abstract])) OR (Perceived Social Supports[Title/Abstract])) OR (Social Support, Perceived[Title/Abstract])) OR (Social Supports, Perceived[Title/Abstract])) OR (Support, Perceived Social[Title/Abstract])) OR (Supports, Perceived Social[Title/Abstract])) | 116,102 | 2:13:13 |
| 2 | (((((((((((((Support, Social[Title/Abstract]) OR (Social Care[Title/Abstract])) OR (Care, Social[Title/Abstract])) OR (Online Social Support[Title/Abstract])) OR (Online Social Supports[Title/Abstract])) OR (Social Support, Online[Title/Abstract])) OR (Social Supports, Online[Title/Abstract])) OR (Support, Online Social[Title/Abstract])) OR (Perceived Social Support[Title/Abstract])) OR (Perceived Social Supports[Title/Abstract])) OR (Social Support, Perceived[Title/Abstract])) OR (Social Supports, Perceived[Title/Abstract])) OR (Support, Perceived Social[Title/Abstract])) OR (Supports, Perceived Social[Title/Abstract]) | 43,527 | 2:09:49 |
| 1 | "Social Support"[Mesh] | 80,491 | 2:07:14 |

Details of search strategy and search results in Web of Science

| # | Search Query | Database | Results | Date Run |
| --- | --- | --- | --- | --- |
| 1 | TS=(Social Support OR Support, Social OR Social Care OR Care, Social OR Online Social Support OR Online Social Supports OR Social Support, Online OR Social Supports, Online OR Support, Online Social OR Perceived Social Support OR Perceived Social Supports OR Social Support, Perceived OR Social Supports, Perceived OR Support, Perceived Social OR Supports, Perceived Social) | Web of Science Core Collection | 477029 | Sat Jan 06 2024 16:22:41 GMT+0800 (中国标准时间) |
| 2 | TS=(turnover intention OR turnover to quit OR turnover to leave OR resignation intention OR turnover intent OR intention to leave OR intent to leave OR intention to quit OR intent to quit OR quit intention) | Web of Science Core Collection | 23620 | Sat Jan 06 2024 16:23:06 GMT+0800 (中国标准时间) |
| 3 | TS=(Nurses OR Nurse OR Personnel, Nursing OR Nursing Personnel OR Registered Nurses OR Nurse, Registered OR Nurses, Registered OR Registered Nurse) | Web of Science Core Collection | 340989 | Sat Jan 06 2024 16:23:24 GMT+0800 (中国标准时间) |
| 4 | #3 AND #2 AND #1 | Web of Science Core Collection | 426 | Sat Jan 06 2024 16:23:44 GMT+0800 (中国标准时间) |

Details of search strategy and search results in Embase

Session Results

.......................................................

No. Query Results Results Date

#37. #16 AND #27 AND #36 62 6 Jan 2024

#36. #28 OR #29 OR #30 OR #31 OR #32 OR #33 OR #34 OR 394,018 6 Jan 2024

#35

#35. 'registered nurse':ti,ab 5,056 6 Jan 2024

#34. 'nurses, registered':ti,ab 260 6 Jan 2024

#33. 'nurse, registered':ti,ab 36 6 Jan 2024

#32. 'registered nurses':ti,ab 12,684 6 Jan 2024

#31. 'nursing personnel':ti,ab 3,457 6 Jan 2024

#30. 'personnel, nursing':ti,ab 46 6 Jan 2024

#29. 'nurses':ti,ab 272,127 6 Jan 2024

#28. 'nurse'/exp 224,652 6 Jan 2024

#27. #17 OR #18 OR #19 OR #20 OR #21 OR #22 OR #23 OR 3,088 6 Jan 2024

#24 OR #25 OR #26

#26. 'quit intention':ti,ab 87 6 Jan 2024

#25. 'intent to quit':ti,ab 118 6 Jan 2024

#24. 'intention to quit':ti,ab 928 6 Jan 2024

#23. 'intent to leave':ti,ab 451 6 Jan 2024

#22. 'intention to leave':ti,ab 861 6 Jan 2024

#21. 'turnover intent':ti,ab 74 6 Jan 2024

#20. 'resignation intention':ti,ab 2 6 Jan 2024

#19. 'turnover to leave':ti,ab 6 Jan 2024

#18. 'turnover to quit':ti,ab 6 Jan 2024

#17. 'turnover intention':ti,ab 883 6 Jan 2024

#16. #1 OR #2 OR #3 OR #4 OR #5 OR #6 OR #7 OR #8 OR 134,501 6 Jan 2024

#9 OR #10 OR #11 OR #12 OR #13 OR #14 OR #15

#15. 'supports, perceived social':ti,ab 6 Jan 2024

#14. 'support, perceived social':ti,ab 41 6 Jan 2024

#13. 'social supports, perceived':ti,ab 6 6 Jan 2024

#12. 'social support, perceived':ti,ab 361 6 Jan 2024

#11. 'perceived social supports':ti,ab 35 6 Jan 2024

#10. 'perceived social support':ti,ab 6,676 6 Jan 2024

#9. 'support, online social':ti,ab 9 6 Jan 2024

#8. 'social supports, online':ti,ab 1 6 Jan 2024

#7. 'social support, online':ti,ab 26 6 Jan 2024

#6. 'online social supports':ti,ab 6 Jan 2024

#5. 'online social support':ti,ab 169 6 Jan 2024

#4. 'care, social':ti,ab 1,394 6 Jan 2024

#3. 'social care':ti,ab 11,831 6 Jan 2024

#2. 'support, social':ti,ab 1,629 6 Jan 2024

#1. 'social support'/exp 120,792 6 Jan 2024

Details of search strategy and search results in CINAHL


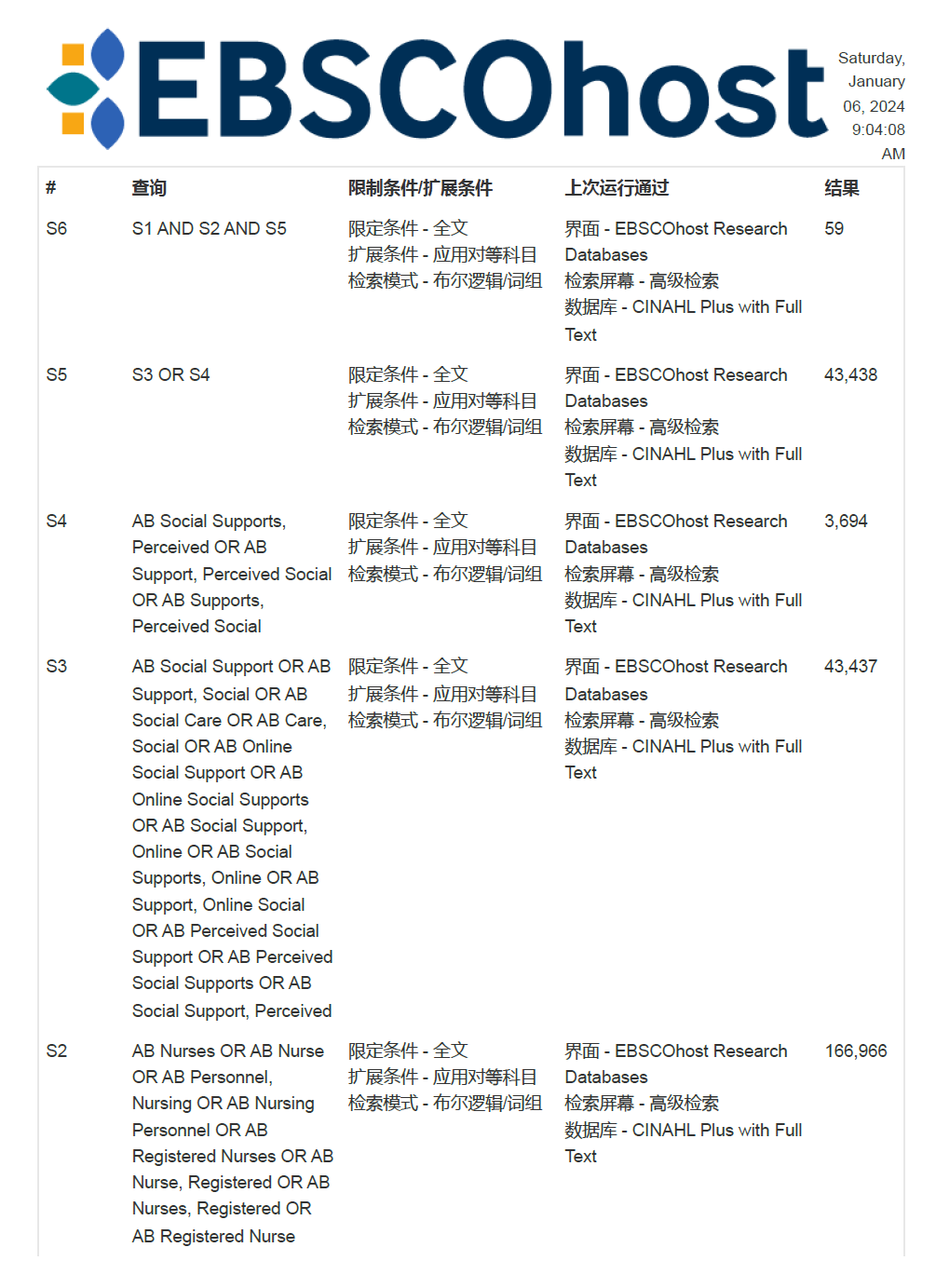


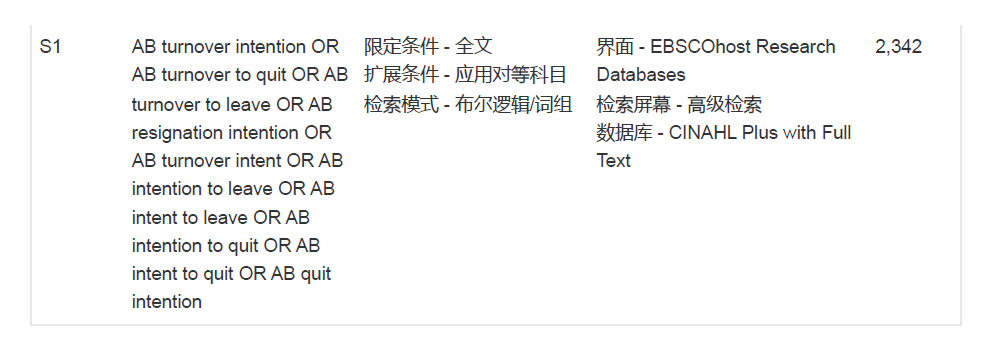

Supplement: Supplementary file 1 [file Data_Sheet_1.docx]
