# Supplementary material for: A systematic review and meta-analysis of the effectiveness of social support on turnover intention in clinical nurses
Source: Front Public Health. 2024 Jun 6;12:1393024. doi: 10.3389/fpubh.2024.1393024 (PMC11187297; doi:10.3389/fpubh.2024.1393024)
Supplement: Supplementary file 2 [file Data_Sheet_2.docx]

Supplementary 2 The quality assessment of included studies

| study | Item1 | Item2 | Item3 | Item4 | Item5 | Item6 | Item7 | Item8 | Item9 | Total |
| --- | --- | --- | --- | --- | --- | --- | --- | --- | --- | --- |
| Schmieder 1996 | Y | U/N | Y | Y | Y | Y | Y | Y | N | 8 |
| Baba 1999 | Y | U/N | Y | Y | Y | Y | Y | Y | N | 8 |
| Pisarski 2006 | Y | U/N | Y | Y | Y | U/N | Y | Y | N | 6 |
| Widerszal-Bazyl 2008 | Y | U/N | Y | Y | N | Y | Y | Y | U/N | 6 |
| van der Heijden 2010 | Y | Y | Y | Y | N | Y | Y | Y | N | 7 |
| Adriaenssens 2011 | Y | U/N | Y | Y | Y | Y | Y | Y | Y | 8 |
| Galletta 2011 | Y | N | Y | Y | Y | Y | Y | Y | U/N | 8 |
| He 2012 | Y | Y | Y | Y | U/N | Y | Y | Y | Y | 8 |
| Fang 2013 | Y | N | Y | Y | U/N | Y | Y | Y | Y | 7 |
| Lei 2013 | N | Y | N | Y | Y | Y | Y | Y | Y | 7 |
| van Dam 2013 | Y | U/N | Y | Y | Y | Y | Y | Y | N | 7 |
| Cai 2014 | N | N | Y | Y | Y | Y | Y | Y | Y | 7 |
| Wu 2015 | Y | Y | Y | Y | Y | Y | Y | Y | Y | 9 |
| Zheng 2015 | N | Y | Y | Y | Y | Y | Y | Y | Y | 8 |
| Zhou 2015 | N | N | Y | Y | Y | Y | Y | Y | Y | 7 |
| Gabel Shemueli 2016 | Y | N | Y | U/N | Y | Y | Y | Y | N | 6 |
| Adriaenssens 2017 | Y | U/N | Y | Y | Y | Y | Y | Y | N | 7 |
| Chen 2017 | Y | N | Y | Y | Y | Y | Y | Y | Y | 8 |
| Tei-Tominaga 2018 | Y | N | Y | Y | Y | Y | Y | Y | N | 8 |
| Xie 2018 | N | N | Y | N | Y | Y | Y | Y | Y | 6 |
| Zhu 2018 | Y | Y | Y | U/N | Y | Y | Y | Y | N | 7 |
| Huang 2019 | N | N | Y | U/N | Y | Y | Y | Y | Y | 6 |
| Wang 2020 | Y | N | Y | U/N | Y | Y | Y | Y | Y | 7 |
| Yeh 2020 | N | N | Y | U/N | Y | Y | Y | Y | Y | 6 |
| Cao 2021 | Y | Y | Y | U/N | Y | Y | Y | Y | N | 7 |
| Cole 2021 | Y | N | N | U/N | Y | Y | Y | Y | Y | 6 |
| Hognestad 2021 | Y | U/N | Y | U/N | Y | Y | Y | Y | N | 6 |
| Meng 2021 | Y | N | Y | Y | Y | Y | Y | Y | Y | 8 |
| Mirzaei 2021 | Y | N | Y | Y | Y | Y | Y | Y | N | 7 |
| Modaresnezhad 2021 | Y | U/N | Y | Y | Y | Y | Y | Y | N | 7 |
| Zhao 2021 | Y | U/N | Y | Y | Y | Y | Y | Y | N | 7 |
| Zhang 2021 | Y | Y | Y | U/N | Y | Y | Y | Y | Y | 8 |
| Wu 2022 | Y | N | Y | Y | Y | Y | Y | Y | N | 7 |
| Xiao 2022 | Y | Y | Y | Y | Y | Y | Y | Y | N | 8 |
| Yu 2022 | N | N | Y | Y | Y | Y | Y | Y | N | 6 |
| Zhang 2022 | Y | Y | Y | U/N | Y | Y | Y | Y | Y | 8 |
| Li 2023 | N | N | N | Y | Y | Y | Y | Y | Y | 6 |
| Xu 2023 | Y | N | Y | Y | Y | Y | Y | Y | Y | 8 |

*Note*. Y, Yes; N, No; U/N, Unclear/Not applicable
