# Supplementary figures and images for: A systematic review and meta-analysis of the effectiveness of social support on turnover intention in clinical nurses
Source: Front Public Health. 2024 Jun 6;12:1393024. doi: 10.3389/fpubh.2024.1393024 (PMC11187297; doi:10.3389/fpubh.2024.1393024)

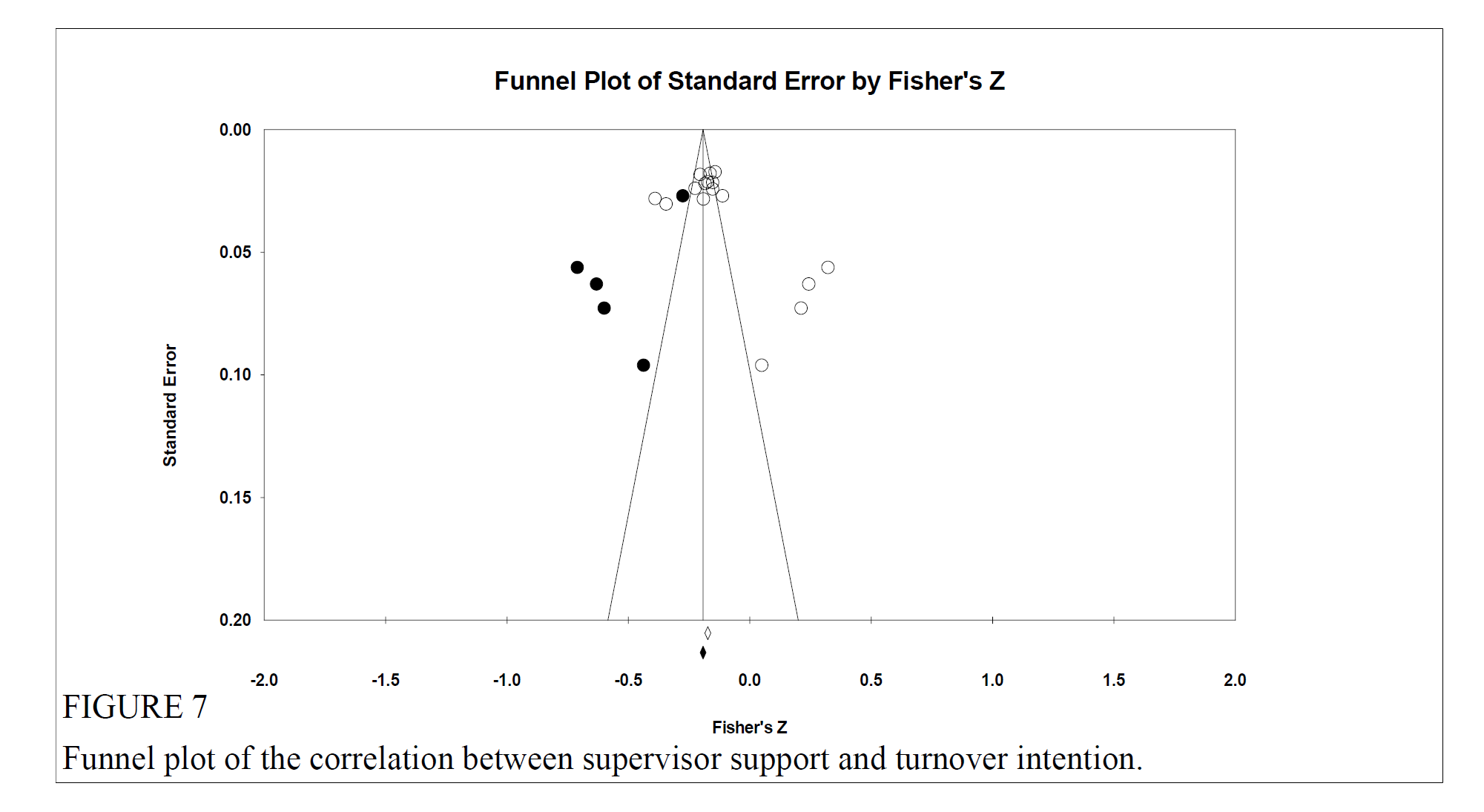


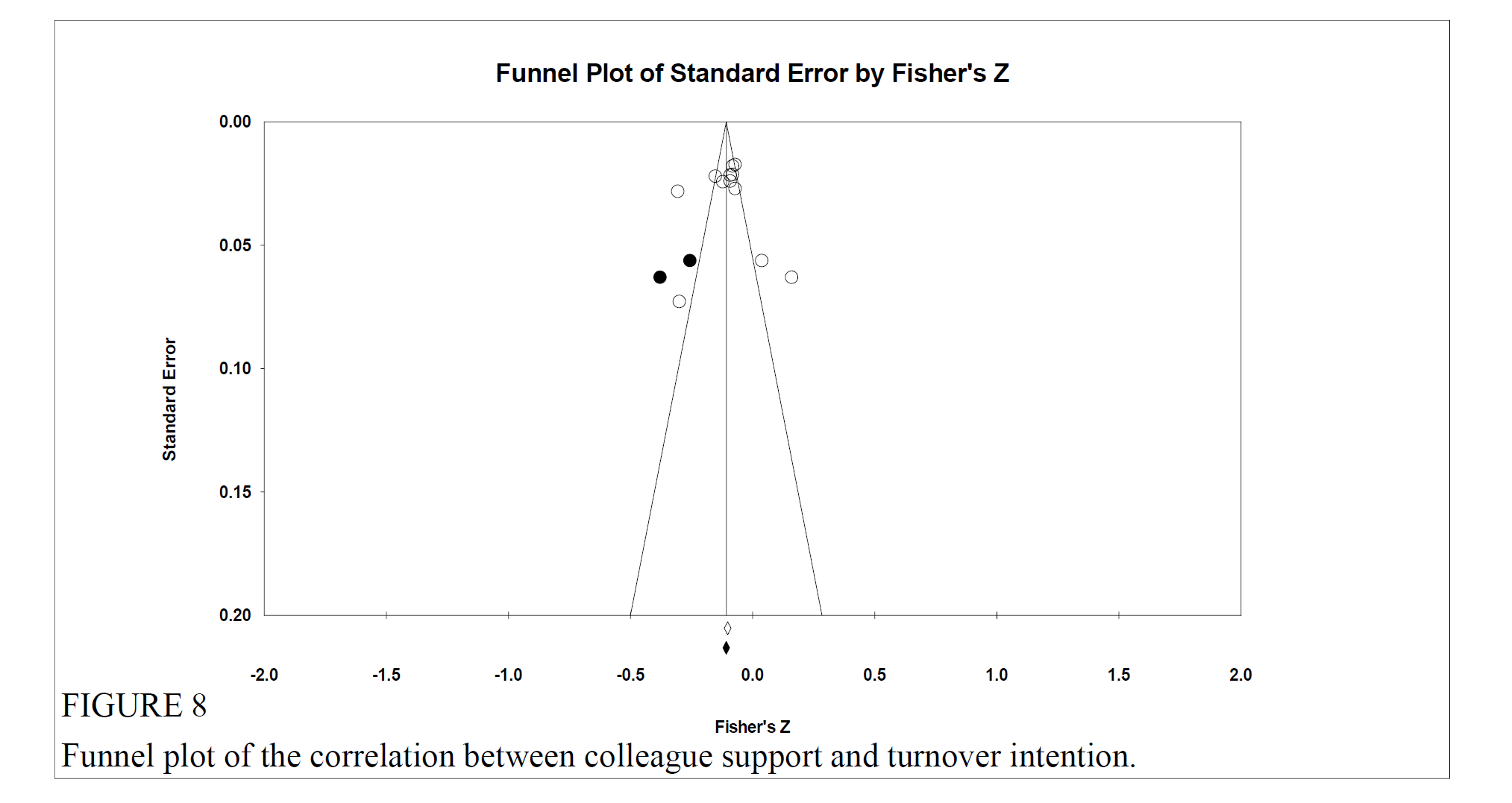


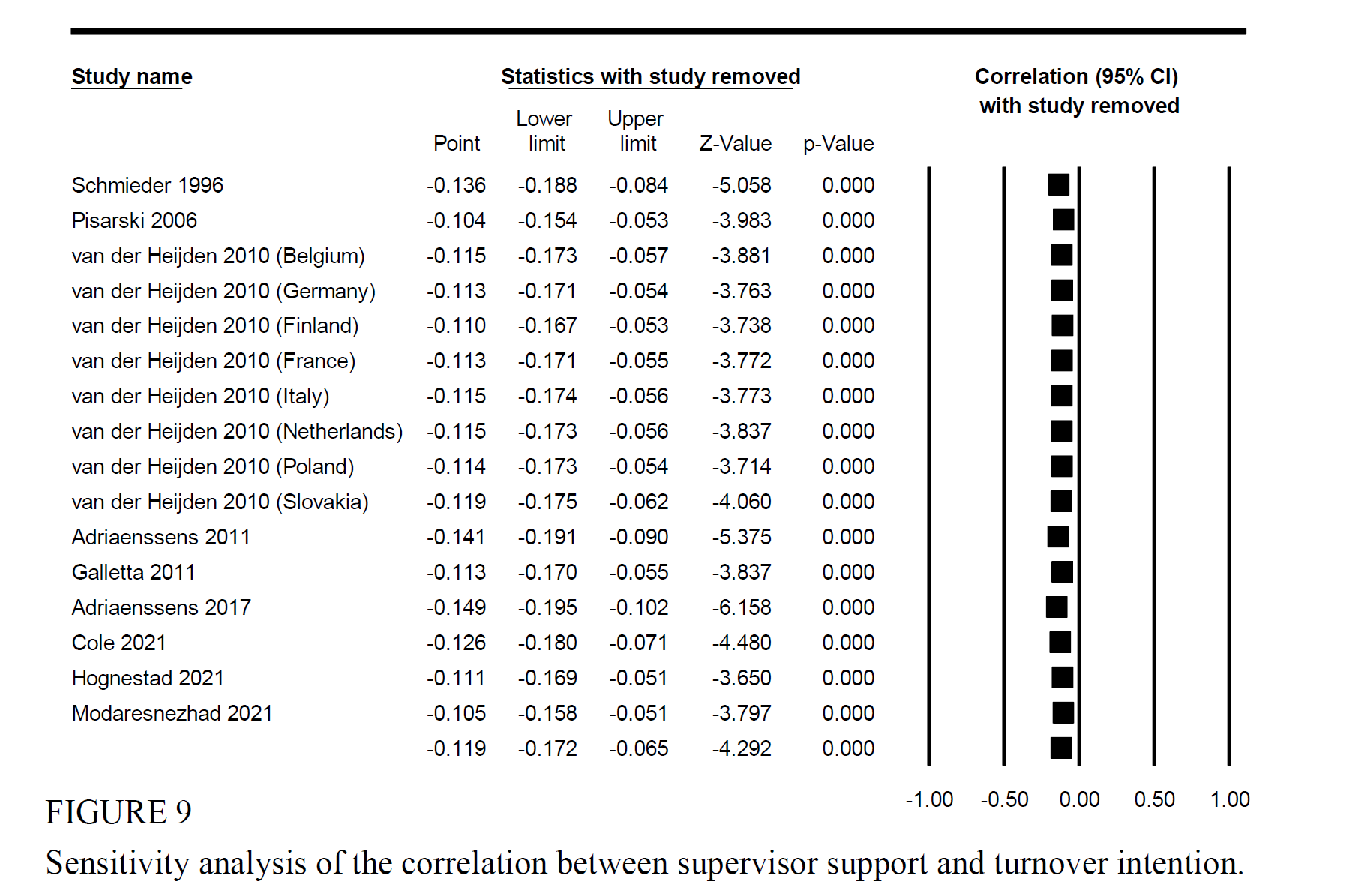


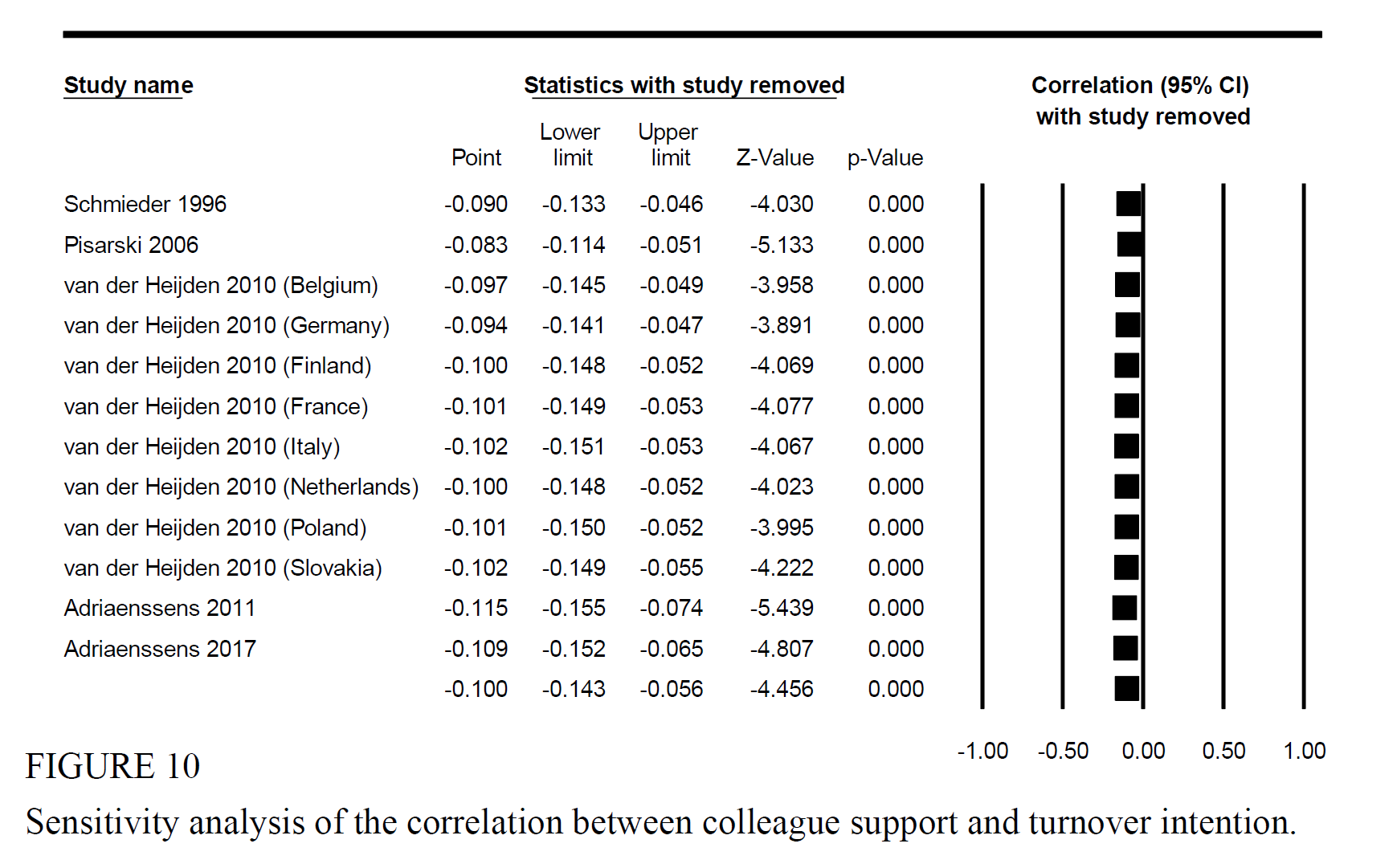

Supplement: Supplementary file 3 [file Data_Sheet_3.docx]
